# Community Health Volunteers in Primary Healthcare in Rural Uganda: Factors Influencing Performance

**DOI:** 10.3389/fpubh.2017.00062

**Published:** 2017-03-29

**Authors:** Yusufu Kuule, Andrew Eric Dobson, Desalegn Woldeyohannes, Maria Zolfo, Robinah Najjemba, Birungi Mutahunga R. Edwin, Nahabwe Haven, Kristien Verdonck, Philip Owiti, Ewan Wilkinson

**Affiliations:** ^1^Church of Uganda Bwindi Community Hospital, Kinkizi Diocese, Kanungu, Uganda; ^2^Aklilu Lemma Institute of Pathobiology, Addis Ababa University, Addis Ababa, Ethiopia; ^3^Institute of Tropical Medicine, Antwerp, Belgium; ^4^Makerere University College of Health Sciences, Kampala, Uganda; ^5^The International Union Against Tuberculosis and Lung Disease, Paris, France; ^6^Academic Model Providing Access to Healthcare, Eldoret, Kenya; ^7^Institute of Medicine, University of Chester, Chester, UK

**Keywords:** community health workers, Bwindi, universal health coverage, sustainable development goals, health system strengthening, operational research, SORT IT

## Abstract

**Introduction:**

Community health volunteers (CHVs) play an integral role in primary healthcare. Several countries rely on CHV programs as a major element in improving access to care and attaining universal health coverage. However, their performance has been heterogeneous and at times context-specific, and influenced by multiple factors. We describe the socio-demographic and workplace characteristics affecting CHVs’ performance in a public health program in rural western Uganda.

**Methods:**

This was a cross-sectional study based on routine program data of CHVs serving the catchment of Bwindi Community Hospital, Kanungu District, South Western Uganda, in 2014 and 2015. Information was collected on individual socio-demographic and workplace characteristics of the CHVs. To assess their work output, we defined study-specific targets in terms of attendance at monthly CHVs’ meetings with community health nurses, households followed-up and reported, children screened for malnutrition, immunization coverage, and health facility deliveries. Frequencies and proportions are reported for characteristics and outputs and odds ratios for study-specific factors associated with overall performance.

**Results:**

Of the 508 CHVs, 65% were women, 48% were aged 35 years and below, and 37% took care of more than the recommended 20–30 households. Seventy-eight percent of the CHVs had ≥80% of pregnant women under their care delivering in health units, 71% had ≥95% of the children on schedule for routine immunization, while 27% screened ≥75% of the children under 5 years for malnutrition. More refresher trainings was associated with better overall performance [adjusted odds ratio (aOR): 12.2, 95% confidence interval (CI): 1.6–93.6, *P* = 0.02] while overseeing more than the recommended 20–30 households reduced overall performance (aOR: 0.6, 95% CI: 0.4–0.9, *P* = 0.02).

**Conclusion:**

Being in-charge of more than the recommended households was associated with reduced performance of CHVs, while more refresher trainings were associated with improved performance. If the CHVs are to remain a strategic pillar in universal health coverage, it is imperative to address those factors known to impact on their performance.

## Introduction

Lay community health workers, often interchangeably referred to as community health volunteers (CHVs), are increasingly recognized as an integral component of the health workforce, especially in low- and middle-income countries (LMICs) (1). Several LMICs invested in CHV programs as a major element of improving access to care and achieving the millennium development goals. In the era of the sustainable development goals (SDGs), CHVs are expected to become even more important as they have been identified as key contributors to universal health coverage, a target on the road to good health and well-being for all (2).

Many LMICs suffer great shortages in the numbers of formal health-care workforce serving the ever-expanding populations. Coupled with limited geographical access to existing facilities or inadequately stocked facilities, these challenges have impacted negatively on health indicators in these countries. Community-based health workers have been crucial in bridging these gaps in provision of equitable healthcare and in expanding essential health services to the community through a range of preventive, promotive, and curative services (2–4).

Several studies have reported on the benefits of CHVs in improving health indicators (2, 5–7). However, the findings have been heterogeneous and influenced by many factors (4, 8), including socio-demographic, political, economic, and workplace environments. Workload, attrition rates, gender roles, level of education, political commitment and employment status, job aides, and physical workplace environment have a significant influence on such workers’ performance (3–6, 9–15). Understanding the contextual factors and enablers that influence the performance of CHVs is relevant for many settings aiming to use them to achieve the SDGs.

## Background and Rationale

In Uganda, the CHV strategy is formally referred to as the Village Health Team. With the guidance of the Ministry of Health and support from several partners, its implementation started in 2002 (8). By January 2015, there were a total of 179,175 CHVs in the country (7). Nationally, CHVs are expected to carry out general tasks in all primary health-care core areas. These include home visits, mobilization of communities to utilize health services, community information management, health promotion and education, management of common illnesses, and follow-up of pregnant women and newborns and follow-up of discharged patients and those on long-term treatment (16).

Assessments of the CHV program in Uganda have shown that CHVs face multiple challenges resulting in poor performance (7, 11, 12). Implementation mechanisms in the country have varied and have been largely steered by government partners, including local and foreign non-governmental organizations, as government funding and resources have dwindled since inception (7). Some of the factors described to improve their performance include support from the community and health facilities, adequate work resources, regular feedback, and supervision (3, 6, 11, 13, 17, 18). Challenges have been attributed to lack of or low remuneration, lack of uniform and regular contact with supervisors, or lack of motivation, high workload, and inadequate skills (3, 12). Supervision and training were often mentioned as facilitating factors, but few studies tested which approach worked best or how these were best implemented (17–19).

Kinkizi Diocese of the Church of Uganda in Kanungu District, South Western Uganda, has been engaging CHVs through Bwindi Community Hospital since 2009. The program has instituted several mechanisms to bolster the performance of the CHVs in its catchment. These have largely focused on addressing the challenges faced by CHVs in their work and providing a conducive work environment including regular supervision and feedback, support from the different health facilities who are supposed to play a role in this, and training to ensure adequate skills while at the same time keeping to the nationally stipulated guidelines (16).

Routine monitoring of the CHVs has indicated varied work outputs, the reasons for which have not been formally and scientifically evaluated. The detailed routine monitoring system at Bwindi Community Hospital provides a unique opportunity to assess the CHV program. This study, therefore, aimed to provide information on these CHV characteristics influencing their work outputs. Specifically, for the rural catchment of Bwindi Community Hospital, we describe (i) the socio-demographic and workplace characteristics of the CHVs, (ii) their work outputs, and (iii) factors influencing their performance. These findings may be relevant to other settings engaging or aiming to engage the CHVs in the drive toward adequate service provision at the community level.

## Essential Elements of the Intervention

In Kinkizi Diocese of Kanungu District, the community health outreach program is run by Bwindi Community Hospital. In addition to the other activities expected of the CHVs which include action for community improvement, infection control during disease outbreaks, family planning in the community, and referral services (16) among others, the hospital specifically monitors them on five outputs: (1) attendance of monthly CHVs’ meetings with community health nurses, (2) household follow-up and reporting, (3) screening and monitoring for malnutrition in under-5 children, (4) immunization coverage, and (5) number of pregnant women in their catchment households delivering in health facilities. Each CHV is expected to visit 25 (±5) households on a monthly basis, carry out health education, and collect indicators on outputs 2–5 above. They are asked to refer children not up-to-date with immunization schedules or alert the community health nurses to follow them up for immunization.

The CHVs are selected in a transparent and participatory manner from those residing within the community, in line with national guidelines (16). Upon recruitment, they are offered basic training using national protocols (20, 21) and provided with a CHV kit containing a bag, nutrition assessment tools, job aides, record books, birth reporting forms, referral cards, and death reporting forms.

The hospital’s community health nurses also conduct biannual 1-day refresher trainings in the CHVs’ villages using the CHV Ministry of Health training manual (20). This is intended to enable the CHVs to maintain their knowledge and skills to mobilize and empower households and community members for health action. The main areas covered in this program include symptoms of childhood illnesses; key indicators for referral of pregnant mothers, children under 5, and other very sick patient in their households; and how to monitor children for malnutrition.

At monthly meetings with a community health nurse from the hospital, held in each village, each CHV submits a pre-structured report. After verification, the report is entered into the hospital database, for further analysis and action. During the same monthly meetings, the hospital’s CHN discusses thematic areas with the CHVs based on their training manual (20) and areas of need as a way of promoting continuous professional development.

In line with government regulations, the CHVs are not paid any salaries (16). However, the hospital motivates them by non-monetary means and in-kind items such as T-shirts, soap, and other small household items. Every 3 months, the respective community health nurses hand over these household items (equivalent to US$ 1.5) at the monthly meetings. The individual CHVs are also formally and informally recognized for their contribution to primary healthcare through their position in the community, contact with health personnel, and more widely in local radio talks and shows.

## Methods

### Study Design

This was a retrospective cross-sectional study carried out between March and September 2016 and based on routinely collected information.

### Setting

Uganda is a land-locked country in East Africa with an approximate population of 40 million people. The country is divided into 112 districts with 84% of the population living in the rural areas and 20% living in poverty (22). Life expectancy was 58 years in 2014. Health coverage in the urban and rural areas is provided by both government and private entities. Primary health facilities play a more prominent role in the rural areas.

The CHV program in Kinkizi Diocese of Kanungu District, South Western Uganda, is present in three of the eight sub-counties of the district—Kayonza, Kanyantorogo, and Mpungu. These three sub-counties have an approximate population of 70,000 people living in 101 villages, which are served by 508 CHVs.

### Study Population

All the 508 CHVs working in the three sub-counties of Kanungu District served by Bwindi Community Hospital in rural western Uganda during 2014 and 2015 were included in the study.

### Data Variables and Sources

The independent data variables were gender, age, highest level of formal education attained, occupation, cell phone ownership, social status, whether a community-based distributor—as in Table 1, length of service of the CHV with the hospital, number of refresher trainings attended, number of households under CHV’s care, and the general topography of the CHV work area. The output variables were (1) the proportions of supervision meetings and trainings attended, (2) households followed-up and reported, (3) children under 5 years screened for malnutrition, (4) immunization coverage, and (5) deliveries conducted in a health unit. All this information was obtained from the hospital electronic database.

**Table 1:** **Socio-demographic and workplace characteristics of the community health volunteers (CHVs) in Bwindi Community Hospital catchment area, Kanungu District, Uganda, 2014–2015**.

Characteristics	*N* (%)
**Gender (*n* = 508)**	
Female	332 (65)
Male	176 (34)
**Age (years) (*n* = 508)**	
≤35	242 (48)
>35	266 (52)
**Level of education (*n* = 501)^b^**	
Primary	334 (67)
Beyond primary	167 (33)
**Occupation (*n* = 505)^b^**	
Subsistence farming	409 (81)
Other income sources	96 (19)
**Owns a cell phone (*n* = 508)**	
Yes	415 (82)
No	93 (18)
**Social status (*n* = 508)**	
Local leader	28 (6)
Non-leader	480 (94)
**Community-based distributor (CBD)^a^ (*n* = 504)^b^**	
Yes	35 (7)
No	469 (93)
**Length of service of the CHV (months) (*n* = 503)^b^**	
≤18 months	36 (7)
19–24 months	467 (93)
**Number of refresher trainings attended (*n* = 503)^b^**	
1–3 trainings	30 (6)
4 trainings	473 (94)
**Number of households (*n* = 497)^b^**	
Large (over 30 households)	184 (37)
Medium (20–30 households)	284 (57)
Small (under 20 households)	29 (6)
**Topography of workplace (503)^b^**	
Hilly	221 (44)
Flat	282 (56)

*^a^A CBD is a CHV with additional training and responsibilities to administer some basic medicines such as anthelmintics and oral antimalarials*.

*^b^The number of CHV is not 508 because some data were missing on these variables*.

### Operational Definitions

For each of the five variables on work output, we defined what was considered “satisfactory” or not. These targets were set specifically for the analyses in this study. The operational definitions are presented in Box 1. Overall work performance was defined as satisfactory if a CHV attained satisfactory status in at least four of the five work output variables.

Box 1 **Study-specific operational definitions for the performance of community health volunteers (CHVs) in Bwindi Community Hospital catchment area, Kanungu District, Uganda, 2014–2015.**

Operational definitions*

1. Meeting attendance: graded as “satisfactory” if the CHV attended ≥75% of the expected monthly meetings.
2. Household follow-up and reporting: graded as “satisfactory” if the CHV visited his/her households, collected, and submitted ≥75% of the expected monthly reports of the followed-up pregnant women, newborn babies, and sick members of his/her catchment households.
3. Malnutrition screening and monitoring: graded as “satisfactory” if the CHV screened and monitored ≥75% of the children less than 5 years of age in his/her catchment households.
4. Immunization coverage: graded as “satisfactory” if the CHV’s catchment area attained ≥95% immunization coverage for children under 5 years of age during the period.
5. Health unit deliveries: graded as “satisfactory” if ≥80% of the pregnant mothers in the CHV’s catchment area delivered in a health facility.

Overall performance: graded as “satisfactory” if a CHV attained “satisfactory” status in at least four of the above five indicators.

*The cut-offs were set specifically for this study.

### Analysis and Statistics

The data were exported from the program electronic database and analyzed using Epi Info 7 (CDC, Atlanta, GA, USA). The CHVs’ characteristics and performance were summarized using frequencies and proportions. To identify factors associated with the performance of the CHVs, binary logistic regression analyses were done and results presented as odds ratios with their 95% confidence intervals (CIs). Multivariable logistic regression analyses were carried out, with overall performance as the binary outcome, to assess strength of the association after correction for possible confounders. All variables with *P* < 0.25 at bivariate level were included in the model unless they were on the same causal pathway. Levels of significance were set at *P* < 0.05.

### Ethics Issues

Permission to carry out the study was obtained from Kanungu District Health Office. Local ethics approval was granted by the Ethics Board of Bwindi Hospital. The study fulfilled the exemption criteria set by the Ethics Review Board (ERB) of Médecins Sans Frontières (MSF), Geneva, Switzerland, for *a posteriori* analyses of routinely collected data and thus did not require MSF ERB review. It was conducted with permission from the MSF Medical Director, Operational Centre Brussels, Belgium. The study was also approved by the Ethics Advisory Group of the International Union Against Tuberculosis and Lung Disease, Paris, France. As this was a record review study, informed consent was not required.

## Results

The socio-demographic and workplace characteristics of the CHVs are shown in Table 1. In total, there were 508 CHVs working in the catchment area of Bwindi Community Hospital. Out of these, 332 (65%) were women, 242 (48%) were aged 35 years and below and 167 (33%) schooled beyond primary level. Two hundred thirteen (43%) of the CHVs were responsible for either more (37%) or less (6%) than the recommended 20–30 households. The reasons that some CHVs had more than 30 households were formation of new households by marriage or immigration and delayed replacement of CHVs.

**Figure 1:**
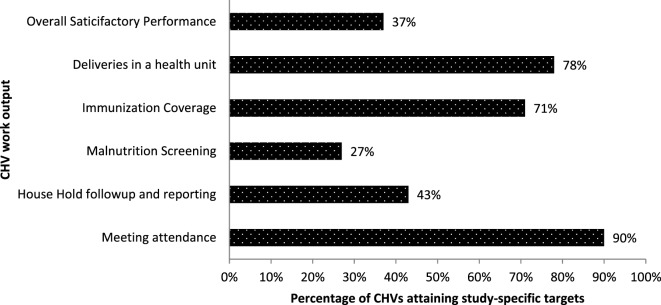
**Proportion of CHVs who achieved set targets on work outputs in Bwindi Community Hospital catchment area, Kanungu District, Uganda, 2014–2015 (*n* = 508)**. CHV, community health volunteer.

Figure 1 shows the proportion of CHVs who achieved the study-specific targets in each of the areas of assessment. In their catchments, 396 (78%) of the CHVs had ≥80% of the pregnant women delivering in health units, 362 (71%) had ≥95% of the children on schedule as regards immunization, while 135 (27%) screened ≥75% of the children under 5 years for malnutrition. One hundred and eighty-seven (37%) of the CHVs attained the study-specific satisfactory overall work performance.

At bivariate analyses, the number of refresher trainings attended and number of households supervised by a CHV was significantly associated with several of their outputs and overall work performance (Table 2). Upon multivariable analysis, more refresher trainings were associated with better overall performance [adjusted odds ratio (aOR): 12.2, 95% CI: 1.6–93.6, *P* = 0.02] while being in-charge of more than the recommended number of households reduced overall performance (aOR: 0.6, 95% CI: 0.4–0.9, *P* = 0.02).

**Table 2:** **Association between individual characteristics and study-specific outputs of community health volunteers (CHVs) in Bwindi Community Hospital catchment area, Kanungu District, Uganda, 2014–2015**.

Characteristic	Meeting attendance	Household follow-up and reporting	Malnutrition screening	Immunization coverage	Deliveries in health unit	Overall performance		
	cOR (95% CI)	cOR (95% CI)	cOR (95% CI)	cOR (95% CI)	cOR (95% CI)	cOR (95% CI)	aOR (95% CI)	*P*-value^b^
**Sex**								
Female	1	1	1	1	1	1	1	
Male	0.9 (0.5–1.6)	1.1 (0.7–1.5)	0.8 (0.5–1.2)	0.9 (0.6–1.4)	0.8 (0.5–1.3)	0.8 (0.5–1.2)	0.7 (0.4–1.0)	NS
**Age (in years)**								
≤35	1	1	1	1	1	1		
>35	1.2 (0.7–2.1)	0.9 (0.6–1.3)	0.9 (0.6–1.3)	1.1 (0.7–1.6)	1.1 (0.7–1.7)	1 (0.7–1.4)	–	–
**Level of education**								
Up to primary	1	1	1	1	1	1	1	
Beyond primary	1.0 (0.5–1.8)	0.7 (0.5–1.0)^a^	1.5 (1.0–2.2)	1.5 (1.0–2.2)	2.5 (1.4–4.2)^a^	1.4 (0.9–2.1)	1.4 (0.9–2.1)	NS
**Occupation**								
Subsistence farming	1	1	1	1	1	1		
Other income Sources	0.9 (0.5–2.0)	0.7 (0.5–1.2)	1.9 (1.2–3.0)^a^	1.0 (0.6–1.7)	1.4 (0.8–2.6)	1.2 (0.7–1.9)	–	–
**Own a cell phone**								
No	1	1	1	1	1	1	1	
Yes	1.4 (0.7–2.9)	0.8 (0.5–1.3)	1.6 (0.9–2.8)	1.1 (0.7–1.8)	1.7 (1.0–2.9)^a^	1.6 (0.9–2.7)	1.5 (0.9–2.6)	NS
**Social status**								
Non-leader	1	1	1	1	1	1		
Local leader	0.7 (0.2–2.1)	0.9 (0.4–1.9)	0.7 (0.3–1.8)	1.5 (0.6–3.8)	0.5 (0.2–1.1)	0.8 (0.4–1.8)	–	–
**CBD**								
No	1	1	1	1	1	1	1	
Yes	2.0 (0.5–8.6)	1.3 (0.6–2.5)	1.2 (0.6–2.6)	1.6 (0.7–4.0)	1.3 (1.0–5.1)	1.9 (0.9–3.9)	2.0 (1.0–4.2)	NS
**Number of refresher trainings**								
1–3 trainings	1	1	1	1	1	1	1	
4 trainings	13.0 (5–33)^a^	2.8 (1.0–7.8)^a^	1.6 (0.6–4.4)	0.7 (0.3–1.7)	2.3 (1.0–5.1)^a^	12.3 (1.6–95)^a^	12.2 (1.6–93.6)	0.02
**Number of households**								
Medium (20–30)	1	1	1	1	1	1	1	
Large (>30)	1.6 (0.8–3.1)	0.6 (0.4–0.9)^a^	0.6 (0.4–1.0)^a^	0.8 (0.5–1.2)	1.3 (0.8–2.1)	0.6 (0.4–0.9)^a^	0.6 (0.4–0.9)	0.02
Small (<20)	0.8 (0.3–2.6)	0.9 (0.4–1.9)	1.9 (0.9–4.2)	1.0 (0.4–2.4)	0.8 (0.3–1.8)	0.9 (0.4–2.1)	0.9 (0.4–2.1)	NS
**Topography**								
Flat	1	1	1	1	1	1	1	
Hilly	1.1 (0.6–1.9)	1.6 (1.1–2.3)^a^	1.4 (1.0–2.1)	0.7 (0.4–1.0)^a^	0.7 (0.5–1.1)	1.3 (0.9–1.9)	1.3 (0.9–2.0)	NS

*^a^P-value <0.05 at bivariate analysis*.

*^b^Only variables with P < 0.25 at bivariate analyses were included in the multivariable model*.

*CBD, community-based distributor: a CHV with additional training and responsibilities to administer some basic medicines such as anthelmintics and oral antimalarials; cOR, crude odds ratio; aOR, adjusted odds ratio; 95% CI, 95% confidence interval; NS, P-value not significant*.

Other variables that were significantly associated with more than one of the work outputs, both before and after adjustment for confounding factors, but not with overall work performance, were the CHV’s formal educational level and village topography. Since the aggregate overall performance measure used was a simple one, these factors may be worth further investigation in subsequent research.

## Discussion

Carried out in the setting of routine programmatic conditions, this study adds to the body of knowledge in the area where CHVs are deemed essential in the attainment of universal health coverage (2, 23). The output of the CHVs was generally satisfactory using the study-specific cut-offs for the assessed outputs. More refresher trainings attended was associated with better overall performance of the CHVs, while oversight of more than the recommended number of households was associated with poorer performance. Only slightly more than half of the CHVs took care of the recommended number of households with the majority of the remaining taking care of more households (See Box 2).

Box 2 **Lessons learnt.**

1. It is possible to monitor community health volunteers (CHVs) work outputs under routine program conditions in rural Uganda.
2. The Bwindi Community Hospital CHVs program puts special emphasis on the importance of regular trainings.
3. The work place performance in this program was heterogeneous but generally satisfactory, with low attrition rate.
4. CHVs work overload threatens work performance.

Strengths of this study are derived from the fact that it assessed all the 508 CHVs working in the catchment area and under the supervision of Bwindi Community Hospital. Conducted under routine program setting, the findings also reflect the operational situation on the ground. Finally, it adhered to the STROBE guidelines in its conduct and reporting (24).

However, there were several limitations:

(1) We were not able to obtain and assess other individual and group characteristics likely impacting the performance of the CHVs, for example, the actual number of hours each CHV works, individual attitudes, the economic status of each CHV, and the community’s perception and reception of the CHVs. Some of these have been identified as being significant factors in motivation of CHVs (13, 14, 17, 18, 25, 26).
(2) Some of the outputs assessed, e.g., delivery in a health unit and immunization coverage can be influenced by factors beyond the CHV input.
(3) Some of the targets set in this study were very high, possibly resulting in an underrating of the performance of some of the CHVs. For example, achieving >95% immunization coverage for children under 5 years of age might normally be seen as very high performance, even though 71% of the CHVs achieved this, and not achieving this would not normally be seen as not “satisfactory.”
(4) The assessment of work output is based on self-reporting by the CHVs who may have been tempted to overestimate their own performance. However, all the information the CHVs generated was discussed with community health nurses during the monthly meetings at the hospital and was only sent to the database after the nurses’ verification and validation.
(5) Finally, problems inherent with the use of routine program data cannot be avoided.

Evaluations of CHVs outputs in multiple settings have yielded heterogeneous results and pointed to the role of contextual factors in the performance of this cadre (1, 8). Individual, group, and program factors have each been shown to influence the performance of CHVs (1, 3, 5, 6, 8–15, 17–19, 25). Factors previously reported to result into improved performance include the female gender, regular supervision and provision of feedback, higher education, the availability of job aides and other tools, and feeling “connected” with the system (3, 5, 6, 9–11, 13, 14, 17, 18).

Our study also showed that number of households a CHV supervises and number of trainings attended were associated with performance. However, it is possible that it is not only the number of trainings attended that resulted in the improved performance, as attending training would also increase contact and communication with the supervising hospital staff and possibly increase the amount of feedback and value that the CHV received. Hence, only an association can be shown, rather than causation. Consequently, we do not know whether these results would be reproduced if the study was conducted in a different setting.

Higher number of households a CHV oversees results into increased workload for these CHVs who are likely working on part-time basis—“volunteering.” More households imply that they have to create more time for these added households and thus time away from their other economic endeavors. Though the program at Bwindi tried to mitigate this by recruiting more CHVs, this clearly did not keep in pace with the growth in number of households. Attrition rates in this program are generally low thus not a contributory factor. CHV programs should thus strive to keep to an optimum threshold of workload to allow family time and other economic endeavors.

Frequent refresher trainings and other engagement platforms such as regular supervisory meetings play an important role in the work output of CHVs. Whether it is the imparted knowledge, the continuous engagement with the formal health sector, the feeling of belonging, the fringe benefits or a combination of such, it is imperative for programs to continually provide an enabling environment to enhance performance of CHVs. It has been reported by Turinawe et al. that although the CHVs in central Uganda were recruited as volunteers they still expected some incentives (26).

The better performance in the shared indicators—deliveries in health units and immunization coverage—points to the role of the other cadres in the output of CHVs. Supervision and monitoring by other health-care workers and the feeling of “part of a whole” are likely to prompt the CHVs to work harder in these areas. The better outputs here may also have been a result of the knowledge that corrective interventions were possible and not “far off.” This is in contrast to screening of children for malnutrition in which interventions are dependent on several other “not easily correctable” factors likely poverty, family size, and socio-economic status of the family and community. However, the role of such “shared” responsibilities and “not easily correctable outputs” in CHVs performance still need further research, as well as the balance between voluntariness, number of work hours, and CHVs’ economic needs.

## Conclusion

If the CHVs are to remain a strategic pillar in universal health coverage, it is important for organizations, national, and local governments and partners to address those factors known to impact on the performance of the CHVs. These include regular supportive supervision and feedback, support from the different health workers who play a complementary role, attending regular refresher courses, and having an appropriate workload.

## Open Access Statement

In accordance with WHO’s open access publication policy for all work funded by WHO or authored/co-authored by WHO staff members, the WHO retains the copyright of this publication through a Creative Commons Attribution IGO license (http://creativecommons.org/licenses/by/3.0/igo/legalcode) which permits unrestricted use, distribution, and reproduction in any medium provided the original work is properly cited.

## Author Contributions

YK conceived the study. YK, DW, MZ, AD, and EW designed the study. YK, RN, PO, and KV helped in data acquisition and analysis. YK, AD, BM, NH, KV, and PO interpreted the findings of the study. YK, PO, and KV drafted the manuscript. DW, MZ, AD, EW, RN, BM, and NH critically reviewed the manuscript. All the authors gave final approval of the published version and agreed to be accountable for the work.

## Conflict of Interest Statement

The authors declare that the research was conducted in the absence of any commercial or financial relationships that could be construed as a potential conflict of interest.
